# Salivary N1-Methyl-2-Pyridone-5-Carboxamide, a Biomarker for Uranium Uptake, in Kuwaiti Children Exhibiting Exceptional Weight Gain

**DOI:** 10.3389/fendo.2019.00382

**Published:** 2019-06-20

**Authors:** Jo Max Goodson, Markus Hardt, Mor-Li Hartman, Hend Alqaderi, Daniel Green, Mary Tavares, Al-Sabiha Mutawa, Jitendra Ariga, Pramod Soparkar, Jawad Behbehani, Kazem Behbehani

**Affiliations:** ^1^Department of Applied Oral Sciences, The Forsyth Research Institute, Cambridge, MA, United States; ^2^Kuwait School Health Program, Kuwait City, Kuwait; ^3^Ministry of Health, Kuwait City, Kuwait; ^4^Faculty of Dentistry, Kuwait University, Kuwait City, Kuwait; ^5^The Dasman Diabetes Institute, Kuwait City, Kuwait

**Keywords:** adolescent obesity, kuwaiti children, N1-Methyl-2-pyridone-5-carboxamide, 2PY, nicotinate metabolism, uranium toxicity, metabolic syndrome

## Abstract

In a longitudinal study of 6,158 Kuwaiti children, we selected 94 for salivary metabolomic analysis who were neither obese (by waist circumference) nor metabolic syndrome (MetS) positive (<3 diagnostic features). Half (43) remained healthy for 2 years. The other half (51) were selected because they became obese and MetS positive 2 years later. In the half becoming obese, metabolomic analysis revealed that the level of salivary N1-Methyl-2-pyridone-5-carboxamide (2PY) had the highest positive association with obesity (*p* = 0.0003, AUC = 0.72) of 441 salivary biochemicals detected. 2PY is a recognized uremic toxin. Also, 2PY has been identified as a biomarker for uranium uptake. Considering that a relatively recent military conflict with documented uranium contamination of the area suggests that this weight gain could be a toxicological effect of long-time, low-level uranium ingestion. Comparison of salivary 2PY in samples from the USA and Kuwait found that only Kuwait samples were significantly related to obesity. Also, the geographic distribution of both reported soil radioactivity from ^238^U and measured salivary 2PY was highest in the area where military activity was highest. The prevalence pattern of adult diabetes in Kuwait suggests that a transient diabetogenic factor has been introduced into the Kuwaiti population. Although we did not measure uranium in our study, the presence of a salivary biomarker for uranium consumption suggests potential toxicity related to obesity in children.

## Introduction

Kuwait is a small country (4.1 million) that lies at the northern end of the Persian Gulf between Iraq and Saudi Arabia. As with many countries of the Middle East, discovery of oil has precipitated a rise in lifestyle disorders such as obesity, hypertension and type 2 diabetes. At the time of this study (2012–2014) the prevalence of adult obesity in Kuwait was 43.4% (Male) to 58.6% (Female) of the population (1), the prevalence of adult hypertension was ~26.3% (2) and the prevalence of type 2 diabetes in adults was about 23.9%, the sixth highest of any country in the world (3).

In the current study, we bring together data suggesting that uranium consumption may have contributed to the development of obesity in Kuwait children. Although uranium does not naturally occur at high levels in Kuwait, an estimated 286 tons of depleted uranium was used in Kuwait (4) as munitions during the Gulf War (1990–1991). Compounds associated with obesity-related diabetes can be identified through the measurement of urine samples in a US population (NHANES 1991–2010). Using inductively coupled plasma mass spectrometry this work has demonstrated that uranium uptake is significantly associated with diabetes (5).

Uranium is a potent renal toxin, with the element accumulating in calcified tissues, livers, and kidneys as a result of both natural and anthropogenic exposure (6, 7). While uranium toxicity can be radiological, uranium chemical toxicity is more acute, and particularly affects the liver, kidneys, and lungs. Chemical toxicity likely involves altered glomerular tubule function or damage, the disruption of cellular ion transport mechanisms, and the inhibition of aerobic oxidative phosphorylation. Renal impairment and uranium poisoning can be caused at dosages as low as 50 ppb to 20 ppm (7–9). Uranium in dust is the largest source of uranium-based radiation exposure in uranium processing facilities (9, 10).

N1-Methyl-2-pyridone-5-carboxamide (2PY) is found at higher concentrations in the serum of patients suffering from renal failure (11, 12). Levels of 2PY in renal tissues of healthy people are typically 1.37 mg/L (± 0.68), whereas concentrations in patients with uremia average 4.02 mg/L (± 3.28) with measurements as high as 7.80 mg/L (± 3.59) (13, 14). In rats, 2PY is one of a number of metabolites whose concentration in urine is associated with prolonged low-dose exposure to uranium (15, 16).

Kuwait has one of the highest obesity and type II diabetes levels in the world for reasons that are not fully understood (1). The objective of this study is to evaluate the development of metabolic disease in Kuwaiti children. We measured blood pressure, height, weight and collected saliva samples from 8,317 children in 2012 and 6,317 again in 2014. From these samples, we selected 94 children with data at both visits, all who were neither obese nor MetS positive at the first visit and approximately half of whom became both obese and MetS positive at the second visit. Saliva samples were tested by metabolomic analysis to determine biomarkers that discriminate these two groups.

## Methods

### Anthropomorphic Measurements

Height measured by stadiometer, weight measured by a calibrated bathroom scale, systolic, and diastolic blood pressure measured by a pediatric automated arm cuff were combined with salivary glucose (17) and salivary high-density lipoprotein cholesterol (HDLC) (18).

### Subject Selection

**Figure 1:**
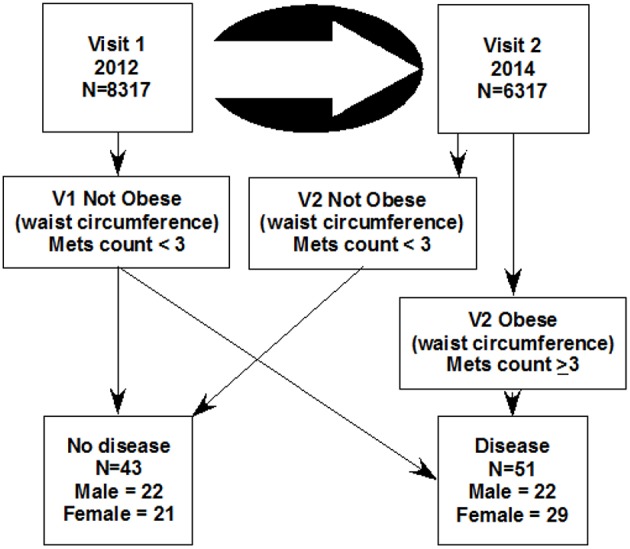
The design of this study which selected 94 children from a longitudinal study of two visits. The metabolic disease category was selected such that 51 were not obese at the first visit and became obese 2 years later at the second visit (the disease group). The 43 control subjects (the healthy group), were selected for not having obesity at either visit. Mets count is the sum of 4 binary metabolomic syndrome characteristics (obesity by waist circumference + high blood pressure + high salivary glucose + low salivary cholesterol).

The study of 8,317 Kuwaiti children (V1, 4th, or 5th grades in 2011–2012) was approved by the Dasman Diabetes Institute Ethical Review Committee in Kuwait. Two years later (2013–2014) 6,317 of the same group were examined using the same methods a second time (V2). Arabic language informed consent was signed by parents/guardians in advance. Subject assent was obtained the day of the visit. Ninety-four children were selected for metabolomic analysis. In the healthy category, 43 children were selected to be metabolic syndrome (MetS) negative at both visits. In the disease category, 51 children were considered healthy on the first visit and developed MetS 2 years later (Figure 1).

For each subject, metabolic disease was evaluated by determining four binary characteristics that define MetS. These included obesity, high blood pressure, and salivary estimates of high blood glucose and low HDLC (19). The diagnosis of MetS positive was defined as having at least three of these four characteristics. Obesity was defined as a waist circumference greater than or equal to the 90th percentile for European children. High blood pressure was defined as either systolic blood pressure >130 mmHg or diastolic blood pressure >85 mmHg. Salivary glucose higher than 1.13 mg/dL, a value determined to be equivalent to 100 mg/dL in plasma (17) was considered hyperglycemic. Salivary HDLC <0.6 mg/dL, a value determined to be equivalent to 40 mg/dL in plasma (18) was designated as low HDLC.

### Saliva Collection

Fasting whole saliva was collected using standard methods (20) in a 15 ml screw-top test tube between 8:30 and 9:00 in the morning before breakfast. Children were asked to rinse and swallow with 15 ml water before collection of 3 ml saliva by drooling. Samples were maintained on ice until centrifuged at 2,800 RMP at 20 min at 4°C.

Supernatant aliquots were transferred to screw-cap 2D barcoded storage tubes (Thermo Scientific) read by a barcode reader (Thermo Scientific VisionMate ST). The barcode was captured with the subject number to a spreadsheet. The sample vials were sealed by a torque-controlled tube capper (Thermo Scientific 8-Channel Screw Cap Tube Capper), placed in a 96-vial rack (Thermo Scientific Latch Rack) and frozen at −80°C. Racks were air-transferred from Kuwait under temperature monitored dry ice (Biocair, Boston MA) to the Forsyth Institute and maintained at −80°C until assay.

### Metabolomic Analysis

Aliquots of saliva supernatants (both the USA and Kuwait) and of plasma samples (only from USA) from each participant (120 μL) were assayed. Relative levels of metabolites were obtained by integrating peaks detected on an untargeted metabolic profiling platform (Metabolon^®^, Durham, North Carolina) which used high-performance liquid chromatography, tandem mass spectrometry, and gas chromatography-mass spectrometry for volatile species (21). Compounds were identified by matching chromatographic retention times and mass spectral fragmentation signatures with reference library data created from authentic standards.

### Statistical Analysis

Analysis was directed toward biochemicals that were most closely related to the development of MetS in the second visit. Anthropomorphic data was evaluated by a two-sample *t*-test. Comparison of salivary biomarkers between diseased and healthy children was computed by the Mann-Whitney *U*-test using the values from the second visit. Analysis by receiver operating curve (ROC) was performed by using the ROC Curve explorer and tester software (22). This software provides the univariate area under the curve (AUC) analysis to predict the transition from health to disease. We computed differences between the USA and Kuwait mass spectrometric analysis of 2PY for each subject from total ion count values divided by the median scaled data for each biochemical. *p*-values were computed for each metabolite. Only those with *p* ≤ 0.01 were included for analysis. In this case, calculated *p*-values were used only to identify those biochemicals most closely associated with obesity not to estimate the true significance level which by Bonferroni adjustment for the 421 biochemicals identified would be *p* ≤ 0.0001. For analysis of covariance, we included the effect of age, sex, five metabolites, and systolic blood pressure.

## Results

We evaluated children who were 9.8 years old at V1 and at 11.9 years old at V2, an elapsed time of ~2 years (Table 1). A significant increase in waist circumference, BMI, systolic blood pressure, and body weight occurred over these 2 years. Height and diastolic blood pressure did not significantly change.

**Table 1:** Anthropometric values of children at V1 and V2.

					**V2–V1 Difference**		
	**Metabolic disease (*n* = 51)**	**Healthy (*n* = 43)**	**Disease-healthy difference**	***p*-value (disease vs. healthy)**	**Metabolic disease**	**Healthy**	***p*-value (V2–V1)**
Age V1(y)	9.8 ± 0.6	9.8 ± 0.6	−0.03	0.8	2.08 ± 0.13	2.1 ± 0.12	0.4
Age V2(y)	11.9 ± 0.6	11.9 ± 0.6	−0.05	0.7			
Waist circumference V1 (cm)	68.4 ± 5.4	60.1 ± 5.8	8.30	<0.0001	20.65 ± 6.19	8.26 ± 7.87	<0.0001
Waist circumference V2 (cm)	89.1 ± 4.6	68.4 ± 9.2	20.68	<0.0001			
BMI V1(Kg/m^2^)	21.0 ± 2.2	17.1 ± 1.8	3.93	<0.0001	5.02 ± 1.78	1.43 ± 1.69	<0.0001
BMI V2 (Kg/m^2^)	26.0 ± 1.5	18.5 ± 2.4	7.51	<0.0001			
Systolic BP V1(mmHg)	112.9 ± 14.9	103.7 ± 15.4	9.23	0.004	16.69 ± 18.01	5.35 ± 22.72	0.008
Systolic BP V2(mmHg)	129.6 ± 11.7	109.0 ± 16.0	20.57	<0.0001			
Diastolic BP V1(mmHg)	77.4 ± 13.0	67.7 ± 10.3	12.39	0.0001	12.39 ±17.17	8.58 ±16.5	0.3
Diastolic BP V2(mmHg)	89.8 ± 11.7	76.3 ± 13.5	8.58	<0.0001			
Height V1 (cm)	138.3 ± 6.7	134.3 ± 7.5	4.08	0.006	13.12 ± 3.70	13.16 ± 3.70	0.95
Height V2 (cm)	151.5 ± 6.7	147.4 ± 9.0	4.03	0.02			
Weight V1(Kg)	40.3 ± 5.6	31.1 ± 6.2	9.19	<0.0001	19.57 ± 4.29	9.5 ± 4.58	<0.0001
Weight V2(Kg)	59.9 ± 6.4	40.6 ± 8.5	19.26	<0.0001			
Male	22	22					
Female	29	21					

*P-values were computed by t-test and indicate the probability that differences between healthy and metabolic disease children were due to chance alone. Mean values ± standard deviations are listed for metabolic disease and healthy subjects. BMI, Body mass index; BP, Blood pressure*.

We identified 421 biochemicals in the saliva samples. Each was tested for their probability of identifying obese children by non-parametric analysis (p) and the area under the receiver operating curve predicting obesity (AUC). By this analysis, N1-methyl-2-pyridone-5-carboxamide (2PY) was identified as the biochemical increasing with obesity most strongly associated with obese children (Table 2). Other metabolites including urate, a sphingomyelin, gamma-glutamylphenylalanine, acisoga, phosphate, threonylphenylalanine, acetylcarnitine, and arginine also increased but to a lesser degree. Elevated uric acid has been associated with MetS, renal, and cardiovascular diseases (23). Elevated sphingomyelin suggests plasma membrane destruction. The dipeptide gamma-glutamylphenylalanine suggests that proteolytic activity is also increased. Acisoga is a spermidine metabolite which has been associated with oxidative stress (24) and the induction of beige adipocytes (25). Phosphate has been previously reported as an obesity-related biochemical (26). An increase of acylcarnitines with obesity, a measure of incomplete fatty acid oxidation, has also been recognized (27). Arginine has not been reported to be associated with obesity. Metabolomic biomarkers decreasing in obese children were mainly lipids suggesting that they arise from intraoral sources rather than from plasma. Previous metabolomic studies indicate that many the biochemicals that decreased in the saliva of obese children were likely the products of oral metabolism (bacterial or mucosal) since none have been found to have a significant correlation between saliva and blood.

**Table 2:** Analysis of salivary metabolite associations with obesity (second visit) for *p* ≤ 0.01 in predicting obesity (Mann-Whitney *U*-test).

**Biochemical**	**Super_Pathway**	**Sub_Pathway**	***p***	**AUC**
**INCREASE**				
N1-Methyl-2-pyridone-5-carboxamide	Cofactors and Vitamins	Nicotinate and Nicotinamide Metabolism	0.0003	0.72
Urate	Nucleotide	Purine Metabolism, (Hypo)Xanthine/Inosine containing	0.0005	0.71
Sphingomyelin (d18:1/24:1, d18:2/24:0)	Lipid	Sphingolipid metabolism	0.002	0.64
Gamma-glutamylphenylalanine	Peptide	Gamma-glutamyl amino acid	0.002	0.56
Acisoga	Amino acid	Polyamine metabolism	0.005	0.67
Phosphate	Energy	Oxidative phosphorylation	0.01	0.65
Threonylphenylalanine	Peptide	Dipeptide	0.01	0.65
Acetylcarnitine	Lipid	Fatty acid metabolism(Acyl Carnitine)	0.01	0.65
Arginine	Amino acid	Urea cycle; Arginine and Proline metabolism	0.01	0.65
**DECREASE**				
1-stearoyl-GPC (18:0)	Lipid	Lysolipid	0.0002	0.73
docosahexaenoate (DHA; 22:6n3)	Lipid	Polyunsaturated Fatty Acid (n3 and n6)	0.001	0.69
N-acetylglycine	Amino Acid	Glycine, Serine and Threonine Metabolism	0.001	0.69
1-stearoyl-2-linoleoyl-GPC (18:0/18:2)	Lipid	Phospholipid Metabolism	0.002	0.69
1-stearoyl-GPE (18:0)	Lipid	Lysolipid	0.002	0.68
1-palmitoyl-2-arachidonoyl-GPC (16:0/20:4)	Lipid	Phospholipid Metabolism	0.003	0.68
oleoylcarnitine	Lipid	Fatty Acid Metabolism(Acyl Carnitine)	0.003	0.64
2'-deoxyinosine	Nucleotide	Purine Metabolism, (Hypo)Xanthine/Inosine containing	0.004	0.65
cholesterol	Lipid	Sterol	0.005	0.67
1-palmitoyl-2-oleoyl-GPC (16:0/18:1)	Lipid	Phospholipid Metabolism	0.008	0.66
isobutyrylcarnitine	Amino Acid	Leucine, Isoleucine and Valine Metabolism	0.009	0.56
1-palmitoyl-2-linoleoyl-GPC (16:0/18:2)	Lipid	Phospholipid Metabolism	0.009	0.66
1-palmitoyl-GPC (16:0)	Lipid	Lysolipid	0.010	0.74
arachidonate (20:4n6)	Lipid	Polyunsaturated Fatty Acid (n3 and n6)	0.01	0.65
sphingomyelin (d18:1/14:0, d16:1/16:0)	Lipid	Sphingolipid Metabolism	0.01	0.60
1-(1-enyl-stearoyl)-2-linoleoyl-GPE (P-18:0/18:2)	Lipid	Plasmalogen	0.01	0.58

*The top ten biochemicals (upper panel) all increased in obese children. Sixteen biochemicals that significantly decreased in the saliva of obese children are listed in the lower panel*.

In this manuscript, we focus principally on 2PY which has been recognized as a uremic toxin associated with metabolic disturbances (28), cancer, and thrombocytopenia (29). 2PY is of interest in this population because of its characterization as a biomarker for uranium uptake (16) and the history of Kuwait which includes a likely source for uranium resulting from a recent (August 1990–February 1991) military conflict (the Gulf War) as a potential source for uranium.

We found 2PY in the saliva of all children that we have analyzed by metabolomics both in the USA and Kuwait, so that differences between individuals appeared as a relative magnitude, not presence or absence. Analysis of the first visit data in the same manner (Table 3) exhibited increased levels of nicotinate ribonucleoside (like 2PY, also a nicotinate metabolite), xanthosine, phenol sulfate, and phosphoenolpyruvate. We also observed reductions related to obesity of sulfate, caffeine, ribitol, phenylacetate, and 4-hydroxyphenylacetate.

**Table 3:** Analysis of salivary metabolite associations with obesity (first visit) for *p* ≤ 0.01 in predicting obesity (Mann-Whitney *U*-test).

**Biochemical**	**Super_Pathway**	**Sub_Pathway**	***p***	**AUC**
**INCREASE**				
Nicotinate ribonucleoside	Cofactors and Vitamins	Nicotinate and Nicotinamide Metabolism	0.00007	0.73
Xanthosine	Nucleotide	Purine Metabolism, (Hypo)Xanthine/Inosine containing	0.004	0.67
Phenol sulfate	Amino Acid	Phenylalanine and Tyrosine Metabolism	0.004	0.67
Phosphoenolpyruvate (PEP)	Carbohydrate	Glycolysis, Gluconeogenesis, and Pyruvate Metabolism	0.006	0.66
**DECREASE**				
Sulfate	Xenobiotics	Chemical	0.001	0.70
Caffeine	Xenobiotics	Xanthine metabolism	0.005	0.55
Ribitol	Carbohydrate	Pentose metabolism	0.01	0.55
Phenylacetate	Amino Acid	Phenylalanine and tyrosine metabolism	0.01	0.65
4-hydroxyphenylacetate	Amino Acid	Phenylalanine and tyrosine metabolism	0.01	0.65

*The top four biochemicals (upper panel) all increased in obese children. Five biochemicals that significantly decreased in the saliva of obese children are listed in the lower panel*.

An analysis of covariance (Table 4) indicates that of those salivary biomarkers that prominently increase with BMI, only 2PY is able to predict elevated BMI with statistical confidence. Systolic blood pressure was also elevated in children with elevated BMI (Table 1). Systolic blood pressure, however, was significantly associated with urate and isobutyl carnitine but not with 2PY. Neither sex nor age were significantly associated covariates with metabolic disease in either case.

**Table 4:** Analysis of covariance in the prediction of BMI and systolic blood pressure by salivary biomarkers (*N* = 94, correlation coefficients = 0.48 for BMI and 0.42 for systolic bp).

	***p*****-Value**	
**Source**	**BMI**	**Systolic BP**
Age	0.60	0.24
Sex	0.27	0.57
N1-methyl-2-pyridone-5-carboxamide (2PY)	0.01	0.12
Urate	0.45	0.04
Acisoga	0.65	0.81
Phosphate	0.53	0.81
Isobutyrylcarnitine	0.19	0.03

We compared the non-targeted metabolomic analysis of 2PY in the saliva of USA children with that of Kuwaiti children in Table 5. In samples from the USA, association with obesity was not significant whereas those of Kuwait were highly significant. Blood samples were taken only from USA subjects. Correlation in 2PY between saliva and blood samples was significant in both study 1 (*r* = 0.50, *p* = 0.0001) and 2 (*r* = 0.73, *p* = 0.00000005). Study 4 with values at both the first and second visits is the study described in this paper. In this group of 94 subjects, 2PY was not associated with obesity in the first visit (*p* = 0.75) but was significantly associated at the second visit (*p* = 0.0003).

**Table 5:** The difference in the normalized spectral abundance of N1-Methyl-2-pyridone-5- carboxamide in saliva samples from obese and not obese children from the USA compared to Kuwait.

				**Obese**		**Not obese**			
**Study**	**Country**	**Average age (y)**	***N***	**Mean**	***N***	**Mean**	**Ntotal**	***p***	**Date of analysis**
1	USA	10.7	22	1.187	46	1.037	68	0.46	2/23/2011
2	USA	12.2	15	1.181	26	0.960	41	0.16	8/16/2012
3	Kuwait	10.1	50	1.008	100	0.675	150	0.0006	3/26/2013
4	Kuwait V2	11.9	51	1.367	43	0.948	94	0.0003	11/3/2015

*Saliva samples from Kuwait exhibited a highly significant difference between obese and those not obese whereas this difference in samples from the USA was not significant by t-test. Study 4 is the study analyzed in this manuscript and described in Tables 1–4*.

**Figure 2:**
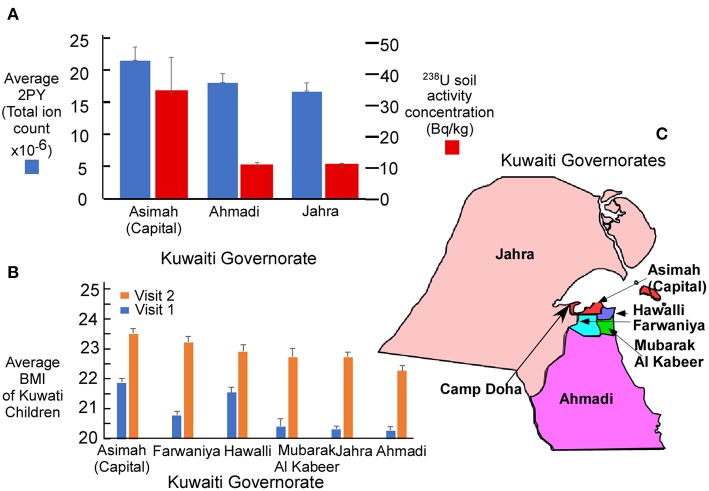
**(A)** Average salivary N1-Methyl-2-pyridone-5-carboxamide (2PY) in Kuwaiti children by mass spectrometry and ^238^U activity concentration (4) in soil following remediation completed after the Gulf War. The principal military facility was located at Camp Doha in the Al Asimah governorate (capital) where both 2PY and ^238^U soil radioactivity were greatest. **(B)** Average BMI of children in each Kuwaiti governorate for V1 (*n* = 8,317) and V2 (*n* = 6,317). **(C)** Relative position of each governorate to Camp Doha. Graphic whiskers in **(A,B)** represent the standard error of the mean for each governorate.

The reported level of soil ^238^U measured following reclamation is compared with salivary levels of 2PY for three Kuwaiti governorates (Figure 2A). Highest levels of both ^238^U and 2PY were in governates close to the U.S. military base (camp Doha). The average BMI of children (Figure 2B) was also highest in those governorates closest to Camp Doha (Asimah, Farwaniya, Hawalli, and Mubarak Al Kabeer) and least in those most distant (Jahra and Ahmadi). The relative location of each of the governates is illustrated in Figure 2C.

**Figure 3:**
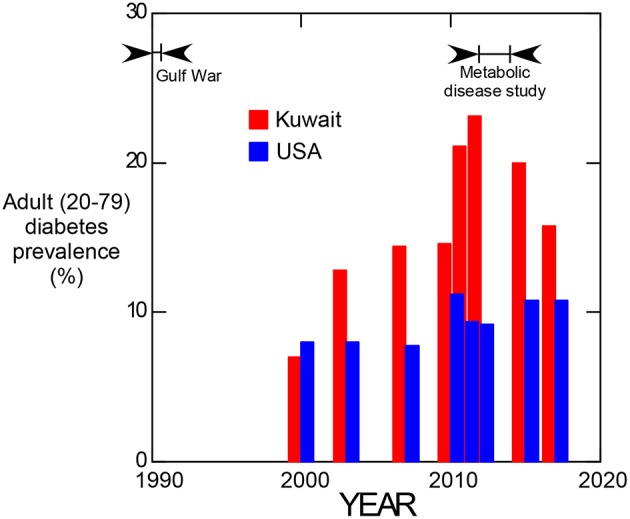
Prevalence of adult (20–79 years old) diabetes in Kuwait and the United States of America from 2000 to 2017. (Data from the International Diabetes Federation, https://www.idf.org/).

Since 2000, adult obesity has been estimated for all countries of the world by the International Diabetes Federation. Comparative prevalence estimates from 2000 to 2017 for Kuwait and the United States are shown in Figure 3. These data suggest that a dramatic increase in the prevalence of diabetes occurred in Kuwait starting at about 2000, reached a maximum in 2012 and has since tended to return to values more comparable to that of the United States.

## Discussion

N1-methyl-2-pyridone-5-carboxamide (2PY) is one of the major metabolites of nicotinamide, a commonly consumed vitamin found in meat, fish, nuts, and mushrooms. 2PY levels are elevated in chronic kidney disease patients where it may act as a uremic toxin by inhibiting poly (ADP-ribose) polymerase-1 (PARP-1) (29). PARP-1 is involved in repair of DNA damage. To our knowledge, 2PY has not been reported to be associated with obesity. Laboratory studies have demonstrated that 2PY inhibits poly(ADP-ribose) polymerase-1 (PARP-1) with an IC50 value of 8 μM (1.2 mg/L).

The appearance of pyridine metabolites (2PY, Table 2, and nicotinate ribonucleoside, Table 3) in saliva with a strong obesity association, suggests that nicotinate and nicotinamide metabolism may be at least in part responsible for these children becoming obese within the 2 years of our study.

We did not find that niacin was consumed to a higher degree in obese children by nutritional analysis (data not shown) and increased niacin consumption is generally associated with weight loss rather than obesity (30). Among other conditions suggested that could trigger this response, consumption of low doses of uranium over 9 months has been found to increase 2PY levels by seven times control in rat urine (16). Elevated levels of 2PY, however, is not uniquely associated with uranium consumption since it was also elevated following high-fat diet protocol without uranium (31).

In support of the uranium hypothesis, between 1990 and 1991, Kuwait was contaminated by an estimated use of 286 tons of depleted uranium during the Gulf War. Measured levels of ^238^U activity concentration in the capital governorate (Asimah) of 13,200 becquerel/kg (Bq/kg)before remediation (4) leaves little doubt of the magnitude of uranium contamination in Kuwait. Geographic distribution (Figure 2C) of salivary 2PY was consistent with the expectation that uranium, salivary 2PY (Figure 2A) and BMI (Figure 2B) were highest in the Asimah governorate where the U.S. military camp was located (Camp Doha) and lower in outlying governorates (Ahmadi and Jahra).

Uranium measured by plasma mass spectrometry, even at the low levels seen in the U.S. population, is a recognized risk factor for diabetes (5). It appears that chemical toxicity of uranium may be of greater concern than radioactivity (32). Urinary uranium was not found to be related to insulin resistance, and it has been suggested that heavy metal toxicity and not radioactivity may be the result of direct β-cell damage (33). Data on occupational exposure indicates that the risk for both kidney disease and cancer are also increased with uranium uptake (34). It should be noted however, that association of uranium exposure with salivary 2PY in humans has not been demonstrated.

It should be noted that association of uranium exposure with salivary 2PY in humans has not been demonstrated, and direct measurement of uranium in salivary or blood tissues would be needed to confirm its role in elevated 2PY levels and obesity. As a further caution, Kuwaiti and American populations may differ in a variety of respects influencing the etiology of obesity. It is therefore possible that factors other than relative uranium exposure are responsible for elevated 2PY levels in obese Kuwaiti children, when these levels are not seen in obese American children.

Considering the temporal values of adult diabetes prevalence in Kuwait (Figure 3), evidence for the occurrence of a transient response to an etiologic factor is suggested. Individuals representing the maximum difference between Kuwait and the United States seen at 2012 would have been 22 years old if born during the Gulf War, indicating that the diabetogenic effect may have been manifest in childhood since diabetes prevalence was not elevated relative to the USA in 2000. Although uranium is implicated by the salivary biomarker data, associated effects such as an influx of westernized diet following the Gulf War also contributed to this effect.

An analysis of covariance for BMI (Table 4) indicated that of the top metabolites only 2PY was significantly associated with obesity. In contrast, elevated systolic blood pressure was significantly associated to both urate and isobutyrylcarnitine.

As part of our study, two validation studies were conducted in Massachusetts and Maine children that collected both saliva and blood of comparable age (17). We found that only in the saliva of Kuwaiti children salivary 2PY was significantly associated with obesity. A critical characteristic of this biomarker is that saliva levels of 2PY were found significantly correlated with plasma levels. This suggests that salivary 2Py levels may serve as a surrogate for plasma 2PY levels.

Elevation of salivary levels of 2PY was only found significant in saliva samples from Kuwait (Table 5). Only the saliva values of studies 3 and 4 demonstrated a significant difference between obese children and non-obese children. In study 4 (the primary study of this manuscript), salivary 2PY levels at V1 when all subjects were neither obese nor MetS positive did not show a significant difference between groups. At V2 the group that became obese and MetS positive had significantly higher levels of 2PY (*p* = 0.0003).

## Conclusions

A juxtaposition of the data indicating that salivary 2PY is higher in Kuwaiti children with the recognition that 2PY is a biomarker for low-level uranium exposure in animals suggests the possibility that obesity in Kuwaiti children could be in part due to uranium toxicity. Uranium toxicity is especially relevant considering that urinary levels have been convincingly associated with diabetes (5).

## Ethics Statement

Although the underlying cause may not be related to uranium consumption, this work demonstrates that elevation of 2PY, a suspected uremic toxin, occurred in these children in association with their exceptional weight gain.

This study was carried out in accordance with the recommendations of

- Nuremberg Code of Ethics
- Belmont Report- Ethical Principles and Guidelines for the Protection of Human Subjects
- World Medical Association Declaration of Helsinki- Ethical Principles for Medical Research Involving Human Subjects

The Committee also takes guidance from applicable international ethical guidelines for biomedical research, such as the International Conference on Harmonization (ICH) guidelines for Good Clinical Practice (GCP) and the Council for International Organizations of Medical Sciences (CIOMS) International Ethical Guidelines for Biomedical Research Involving Human Subject and Epidemiological Studies. The study was approved by the Dasman Diabetes Institute Ethical Review Committee. It was also reviewed and approved by the Forsyth Institutional Review Board. Written informed consent was obtained from parents or guardians of each child before initiation of the study. In addition, a signed assent form was obtained from each child at the time of examination.

## Author Contributions

The study was designed by JG, JB, and KB. M-LH, JG, HA, MT, JA, and PS directed the work. HA, JA, AM, and PS directed clinical research activity in Kuwait. MH and DG reviewed and critiqued the metabolomic analysis portion of the study.

### Conflict of Interest Statement

The authors declare that the research was conducted in the absence of any commercial or financial relationships that could be construed as a potential conflict of interest. The handling Editor declared a shared affiliation, though no other collaboration, with one of the authors KB.
